# Key Performance Indicators for Service Robotics in Senior Community-Based Settings

**DOI:** 10.3390/healthcare13070770

**Published:** 2025-03-30

**Authors:** Yunho Ji, Joonho Moon, YoungJun Kim

**Affiliations:** 1 College of Business Administration, Kangwon National University, Chuncheon 24341, Republic of Korea; yunho.ji@kangwon.ac.kr (Y.J.); joonhomoon@kangwon.ac.kr (J.M.); 2 Graduate School of Management of Technology, Korea University, Seoul 02841, Republic of Korea

**Keywords:** service robotics, senior community, gerontechnology, key performance indicator (KPI), Mixed Methods Research (MMR)

## Abstract

**Objectives**: This study aims to develop performance indicators for service robotics in senior community-based environments and analyze their impact on independent living and quality of life for older adults. **Methods**: To achieve this, a sequential exploratory design within the Mixed Methods Research (MMR) framework was employed, integrating qualitative research (Focus Group Interview, FGI) and quantitative research (Analytic Hierarchy Process, AHP). The FGIs were conducted with a panel of six experts over three rounds, leading to the identification of six key performance indicators (KPIs) for service robotics in senior communities: Technical Performance, User-Centered Performance, Social and Psychological Impact, Ethical and Safety Performance, Economic and Operational Performance, and Service Efficiency. Following this, the AHP analysis was conducted with a final sample of 29 participants from an initial 32 respondents. **Results**: The AHP analysis results revealed that Technical Performance (rank 1, 0.256) was the most critical factor, followed by User-Centered Performance (rank 2, 0.205) and Social and Psychological Impact (rank 3, 0.167). These findings suggest that enhancing a user-friendly, intuitive UI/UX is essential for ensuring ease of use by older adults. Additionally, while Ethical and Safety Performance (rank 3, 0.139), Economic and Operational Performance (rank 4, 0.126), and Service Efficiency (rank 5, 0.105) had relatively lower importance scores, the study highlights the necessity of establishing optimized systems through ethical and safety standards and emphasizes that real-time monitoring systems play a crucial role in enhancing operational efficiency. **Conclusions**: Enhancing service robotics performance requires prioritizing technical capabilities and user-centered design, along with ethical standards and real-time monitoring. This study proposes a structured evaluation framework to support more effective robotic solutions in senior care environments.

## 1. Introduction

As global aging accelerates, ensuring independent living and maintaining the health of the elderly has become a significant social challenge [1]. In particular, Continuing Care Retirement Communities (CCRCs) and other senior communities are expanding, especially in developed countries, to support the physical and mental well-being of older adults and enhance their quality of life [2]. Senior communities are evolving from mere residential spaces into integrated and innovative environments that focus on health management, social interaction, and improved living standards for the elderly [3]. Key trends in senior communities include digital transformation, wellness-oriented design, sustainability, smart technology integration, and community-based care services [4,5]. In this context, service robotics is gaining attention as a key solution for rising elderly care needs and efficient senior community management [6].

Senior communities are structured into different levels of care, including independent living, assisted living, and long-term care, allowing for tailored services according to the needs of residents [7]. Simultaneously, technological advancements and digital transformation are accelerating the role of service robotics (Robot as a Service, RaaS) within these communities [8]. Service robots contribute to health monitoring, daily assistance, emotional support, and facility management efficiency, ultimately becoming a core technology for building sustainable, age-friendly communities [9,10].

Moreover, the rapid aging of the population and increasing demand for care services further emphasize the necessity of service robotics [11]. The global rise in the elderly population has led to a shortage of caregivers, imposing significant burdens on individuals and society. As a solution, service robotics can take over repetitive and physically demanding caregiving tasks, alleviating the burden on human caregivers while enabling more personalized and emotionally supportive care [12,13]. The integration of service robotics within senior communities is driven by factors such as an aging population, a shortage of caregivers, and the need for continuous health monitoring and personalized care [14]. Additionally, technological advancements in AI, IoT, and automation have enabled service robots to provide a wide range of functions, including daily assistance, mobility support, health monitoring, social interaction, and facility management [9,10,15].

Gerontechnology has emerged as a new interdisciplinary field that focuses on supporting the physical and cognitive functions of older adults while creating safer and more convenient living environments [16]. In this context, service robotics serves as a key technological application of gerontechnology and has been widely studied in technology and business management (TBM) and social welfare disciplines. In the technology and business management (TBM) field, research has focused on technological advancements and innovation strategies, market feasibility and business models, optimal adoption and operational management, sustainability, and ethical considerations related to service robotics [4,5,7]. Meanwhile, in the social welfare field, studies have explored the impact and limitations of service robotics on elderly health and well-being, social inclusion, caregiving service improvements, and ethical issues [3,10]. However, previous research has primarily examined service robotics from isolated perspectives, lacking a comprehensive framework for assessing its actual effectiveness in senior communities. Investigating the key factors that enable senior communities to evolve into more intelligent and interconnected ecosystems through service robotics is crucial.

This study aims to develop key performance indicators (KPIs) for service robotics in senior communities and to establish a systematic evaluation framework by integrating perspectives from technology, business management, and social welfare. Using a mixed-method approach, it combines Focus Group Interviews (FGIs) to gather expert insights and the Analytic Hierarchy Process (AHP) to quantify key performance indicators. The findings will identify value creation mechanisms and propose optimal operational strategies for implementing service robotics in senior communities.

## 2. Theoretical Background

### 2.1. Service Robotics as Gerontechnology

Gerontechnology studies the interaction between aging individuals and technology, aiming to develop technologies that promote the health, safety, independence, and social engagement of older adults [16,17]. Within this framework, service robotics represents an engineered mechanism that incorporates age-friendly technology tailored to the needs of older adults [18,19]. Service robots are engineered devices specifically designed for direct interaction with humans, performing a range of tasks including domestic chores (e.g., cleaning, cooking, and mobility assistance), healthcare-related services (e.g., medication reminders, health monitoring, and physical rehabilitation support), and socio-emotional support (e.g., companionship, communication facilitation, and cognitive stimulation), thereby enhancing the quality of life, autonomy, and well-being of users, particularly older adults [11,20]. Moreover, the implementation of such robots may mitigate caregiving burdens, alleviate social isolation among older populations, and support aging in place through personalized and adaptive interventions, thus contributing to sustainable elderly care models [21,22]. In aging societies, these robots play a crucial role in enhancing seniors’ quality of life and reducing long-term social burdens [12,23]. That is, service robotics is designed based on the principles of gerontechnology, providing optimized automation systems and interfaces that allow older adults to use technology more conveniently and safely [24,25].

From the viewpoint of the Health Belief Model (HBM), the utilization of service robotics by older adults can be examined [26]. Seniors may recognize a heightened vulnerability to health concerns, including falls, chronic illnesses, and social isolation, which service robots can help address by providing real-time monitoring and support [11,27]. The perceived severity of these risks, such as the potential consequences of unmanaged health conditions or a lack of social engagement, further reinforces the need for technological intervention [28]. Aging is accompanied by declining physical function, increased chronic diseases, and a heightened risk of emergency situations, necessitating more effective healthcare management and medical services [29]. Service robotics incorporates artificial intelligence (AI) and the Internet of Things (IoT) technologies to facilitate continuous health monitoring, transmitting essential medical information to healthcare providers and caregivers. This technological integration supports proactive health management strategies and ensures rapid responses to medical emergencies [30,31]. In particular, service robotics utilizes wearable biosensor devices and integrated telemedicine platforms to provide tailored healthcare support for older adults, especially those needing chronic disease management and timely emergency interventions [32].

Additionally, beyond physical health, emotional stability and social bonding are crucial factors in maintaining quality of life for seniors [33]. However, due to limited mobility, the loss of spouses or friends, and reduced social roles, older adults are at an increased risk of social isolation and depression [34]. To mitigate these challenges, service robotics facilitates emotional interaction and social connectedness, contributing to the psychological well-being of older adults [35]. AI-based conversational robots utilize emotion recognition technology to understand seniors’ feelings and provide customized interactions, assisting with daily conversations and offering emotional stability [36,37].

### 2.2. Digital Transformation of Senior Communities

A senior community is a residential environment designed to enable older adults to maintain a safe and independent lifestyle while receiving necessary medical and welfare services [38]. These communities have evolved beyond traditional senior housing facilities into integrated spaces that combine social interaction, healthcare, life support, and leisure activities [39]. Internationally, the Continuing Care Retirement Communities (CCRCs) model serves as a representative case, offering a multi-tiered support system that includes independent living, assisted living, and long-term care, depending on the physical and mental health status of seniors [40]. This multi-layered support model allows for the gradual provision of care services tailored to the individual health conditions of seniors.

Senior communities are designed to enhance the quality of life for older adults by providing an integrated living environment that includes healthcare management, social interaction, life support, and leisure activities [41,42]. These communities are evolving into sustainable housing and medical models beyond simple senior residences [4]. Accordingly, senior communities incorporate healthcare and medical support systems, promotion of social interactions and community-based care services, smart environments and self-sufficiency technologies, and sustainable operations with eco-friendly designs [43,44]. The application of artificial intelligence (AI), the Internet of Things (IoT), big data, and telemedicine plays a critical role in the digital transformation of senior communities [45].

Digital transformation is an essential process to maximize the operational efficiency of senior communities and improve residents’ quality of life [46]. It facilitates the adoption of smart healthcare, automated life support, and digital platforms for enhanced social interaction. In an aging society, the digital transformation of senior communities goes beyond simple technological adoption; it actively supports active aging, health maintenance, and social relationship formation [47,48]. This transition can be effectively explained through Social Network Theory. Social Network Theory states that the structure and interactions within personal relationships influence health, psychological well-being, and social participation [49]. Physical activities such as sports, yoga, and swimming utilizing virtual reality (VR) or wearable devices support seniors in maintaining muscle strength and cardiovascular health [50,51]. Participation in group exercise programs or sports clubs based on digital platforms not only aids in health management but also promotes social interaction, contributing to emotional stability and motivation [52].

From the perspective of Environmental Gerontology, the development of personalized leisure services using advanced technology for seniors requires a user experience (UX)- and accessibility-oriented design [53]. When the person–environment fit (P-E Fit) is optimized, seniors experience enhanced activity and independence [54]. In other words, when digital technology is tailored to the physical and cognitive characteristics of older adults, an environment that facilitates their participation in leisure activities is established [55]. Leisure technologies designed with a focus on UX and accessibility can help seniors lead more vibrant and healthy lives, significantly improving their quality of life through customized leisure services [56,57]. This suggests that technology adoption should not be limited to simple implementation but must be designed in a way that enables easy usage, leading to increased engagement and improved quality of life for seniors.

## 3. Research Methodology

### 3.1. Mixed Methods Research (MMR)

This study is designed to develop key performance indicators for service robotics in senior communities by applying the Sequential Exploratory Design, a research methodology within the Mixed Methods Research (MMR) framework proposed by Creswell and Creswell (2005) [58]. The research process is structured as illustrated in Table 1, integrating qualitative and quantitative research methods in a sequential manner.

This research methodology follows a typical exploratory approach which consists of two main phases. In Phase 1 (qualitative research), the study identifies key concepts related to the research topic, extracts relevant variables, and develops a theoretical model through a participant-centered approach. This phase lays the groundwork for understanding the research problem by exploring fundamental themes and constructing a conceptual framework. In Phase 2 (quantitative research), the concepts identified in the qualitative phase are measured and the relationships between variables are analyzed. The findings are then validated through statistical analysis, ensuring the empirical robustness and generalizability of the research outcomes. A key advantage of the MMR approach is its ability to compensate for the limitations of quantitative research. Quantitative studies often struggle to identify additional causal variables beyond pre-defined ones. However, by incorporating qualitative research, this study enables exploration of potential variables from the participants’ perspective, providing a more comprehensive basis for addressing research questions [59].

Given the lack of empirical studies on gerontechnology-integrated service robotics, this study adopts the following:

- Phase 1—Focus Group Interviews (FGIs) are used to derive key performance indicators (KPIs) for service robotics in senior communities;
- Phase 2—The Analytic Hierarchy Process (AHP) is used to evaluate the relative importance of the identified key performance indicators.

This methodological approach is an effective means to establish sustainable principles and guidelines for key performance indicators while also enhancing the positive perception of service robotics in senior communities.

### 3.2. Qualitative Research Utilizing Focus Group Interviews (FGIs)

This study conducted Focus Group Interviews (FGIs) to engage experts in relevant fields in an in-depth discussion on the concept, definition, and practical implications of service robotics in senior communities, with the objective of developing key performance indicators. A FGI is a qualitative research method that collects data through participant interaction to gain insights into perceptions and opinions on a specific topic [60]. Unlike traditional individual interviews, FGIs are particularly suitable for in-depth exploration of a focused subject, involving a homogeneous group that facilitates discussions aligned with the research objectives [61].

For participant selection, homogeneity was considered to ensure smooth communication and information exchange, while segmentation was applied to incorporate diverse perspectives from various expertise areas [62]. Accordingly, to obtain a comprehensive and multidimensional perspective, this study carefully selected six experts from diverse fields. The expert panel included professionals from gerontology and social welfare, robotics engineering, software development, senior welfare service planning, and legal and security analysis. This diverse composition ensured that the study captured a balanced understanding of both the technical and social dimensions of service robotics in senior communities. By integrating insights from these varied disciplines, this research aimed to develop key performance indicators that are both practically relevant and theoretically grounded. The composition of the participants is detailed in Table 2.

Prior to conducting the FGIs, the ethical validity of this study was considered with respect to the following two issues. First, the FGIs must comply with fundamental research ethics principles by ensuring participants’ autonomy, confidentiality, and minimal harm. Second, methodological rigor and ethical responsibilities should be balanced by promoting group dynamics while avoiding infringement upon participants’ autonomy or undue pressure. To address these issues, the authors reviewed the Institutional Review Board (IRB) exemption self-assessment checklist provided by the affiliated institution and obtained signed consent forms for personal information collection and usage from all six FGI participants. Additionally, the purpose, significance, methodology, procedures, and potential applications of the study were openly shared with all participants via email in advance, thoroughly addressing ethical considerations.

The FGI process was conducted in three phases from October 10 to November 21, 2024. All recorded data were transcribed and organized for analysis in the first session. The transcribed data were analyzed through coding and categorization in the second session. The Constant Comparison Method was applied to validate the categorized themes in the third session [63]. Through this systematic FGI process and iterative analysis, this study refined the key performance indicators for service robotics, ensuring their empirical validity and applicability within senior communities.

### 3.3. Quantitative Research Using AHP

The Analytic Hierarchy Process (AHP) is a methodology designed to systematically and quantitatively solve complex decision-making problems [64]. It is one of the widely used methods for multi-criteria decision making (MCDM) and is particularly valued for its ability to measure logical consistency. As a result, the AHP has been applied across various fields, including management, marketing, policy-making, and project management [65].

The field of service robotics focuses on robots designed to assist humans or provide specific services. Given its nature, it requires segmentation based on the purpose of physical resources and activities and an active, adaptive approach. Therefore, AHP analysis in the service robotics sector proves to be a useful decision-making tool, as it can systematically evaluate complex factors such as hardware configurations, software algorithms, and user requirements.

To reflect the characteristics of the service robotics domain, this study employed both convenience sampling and snowball sampling, and conducted the survey from December 11 to December 20, 2024. The field of service robotics requires specific technical expertise, making it challenging to rely solely on random sampling [66]. Therefore, snowball sampling was employed alongside, utilizing existing expert networks to recruit additional respondents. This approach allowed for the inclusion of experts from diverse backgrounds, ensuring a more balanced evaluation and minimizing bias toward any particular group [67]. The AHP survey participants were selected from professionals with substantial expertise in the field of service robotics. The eligibility criteria included the following: Professionals with at least 10 years of industry experience in robotics-related fields, professionals with a master’s degree in a related field and more than 5 years of experience, and experts with a doctoral degree working in academia or research institutions specializing in service robotics. The sample size of this study was determined based on methodological guidelines and comparable precedent studies involving the Analytic Hierarchy Process (AHP). According to Saaty (1994) [68], the AHP typically does not require large sample sizes due to its reliance on expert judgment and pairwise comparisons, and generally an expert panel of around 30 participants is considered appropriate [69]. Therefore, this study selected a sample size within this recommended range to guarantee methodological rigor and analytical validity. A total of 32 respondents participated in the survey. However, following the standard consistency ratio (CR) threshold of 0.2 commonly applied in social sciences, three responses exceeding this limit were excluded [70]. Consequently, 29 responses were retained for the final analysis. The demographic characteristics of these 29 respondents are presented in Table 3.

The statistical analysis for this study was conducted using the Social Science Research Automation (SSRA) system, which operates on a cloud-based infrastructure. The survey was administered via the ssra.or.kr website. The survey included a pairwise comparison table based on a 17-point scale, with detailed descriptions for each variable to help respondents better understand the evaluation criteria. In this study, the AHP analysis of service robotics produced the following results: the Substitutability Index, the Integrated Geometric Mean Matrix, and the Importance of Each Evaluation Criterion, all calculated collectively.

## 4. Research Results

### 4.1. FGI Analysis Results

To explore key performance indicators for service robotics in senior communities, which is the primary objective of this study, three rounds of Focus Group Interviews (FGIs) were conducted with relevant experts and practitioners.

The first FGI was conducted based on the questioning techniques and processes suggested by Krueger and Casey (2015) [71]. During this session, participants exchanged opinions and insights about service robotics, discussed various cases and experiences related to the digital transformation of senior communities, and explored different types of services, social and psychological benefits, and policy support and legal considerations.

The second FGI analyzed the transcribed content from the first session. The primary focus of this stage was to identify potential key performance indicators for service robotics in senior communities. Participants then grouped similar indicators and categorized them based on the purpose of the robots and the specific characteristics of the operational environment.

The third FGI aimed to finalize the names and definitions of the categorized key performance indicators to ensure they accurately reflect the core functionalities of service robotics. Additionally, participants provided feedback on potentially missing indicators and suggested new ideas for future development.

The three rounds of FGIs (Focus Group Interviews) resulted in the consolidation and analysis of expert opinions, which led to the classification of key performance indicators into the following six categories; Technical Performance, User-Centered Performance, Service Efficiency, Economic and Operational Performance, Social and Psychological Impact, and Ethical and Safety Performance. These six categories provide a structured and comprehensive framework that effectively addresses the core performance aspects of service robotics in senior communities. Moreover, they will serve as the hierarchical factors for the AHP (Analytic Hierarchy Process) analysis conducted in this study.

The key details derived from the transcribed FGIs discussions are presented in Table 4 below.

### 4.2. AHP Verification

#### 4.2.1. AHP Model Structure

After completing the qualitative research process using FGIs (Focus Group Interviews), this study conducted an AHP (Analytic Hierarchy Process) analysis to justify the interpretation of the research findings and ensure their objectivity and reliability. The AHP model in this study is composed of six core domains, which include Technical Performance, User-Centered Performance, Service Efficiency, Economic and Operational Performance, Social and Psychological Impact, and Ethical and Safety Performance.

**Figure 1:**
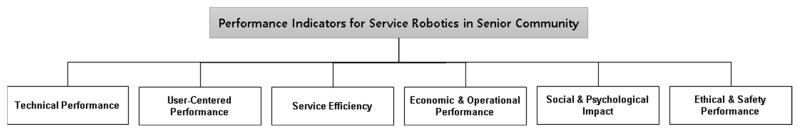
AHP analysis model.

The six key performance indicators derived from the FGIs in this study were operationally defined for the AHP analysis, as shown in Table 5. Additionally, to analyze the priority of these key performance indicators, the AHP model was structured into two hierarchical levels. The analytical model proposed in this study is presented in Figure 1.

#### 4.2.2. AHP Analysis Results

The AHP calculates the consistency index (CI) and consistency ratio (CR) to ensure the consistency of the results. These measures help assess the reliability of the pairwise comparisons and determine whether the judgments made by respondents are logically coherent. The consistency check in the AHP was performed using the following formulas:

Calculation of the maximum eigenvalue (λmax):

$$\lambda \;max=\sum (Wi\times \sum \frac{Aij}{Wj})$$

where, Aij represents the elements of the pairwise comparison matrix. Wi denotes the elements of the weight vector.

Calculation of the consistency index (CI):

$$CI=\frac{\lambda \;max-n}{n-1}$$

where, is the size of the matrix (i.e., the number of compared elements)

Calculation of the consistency ratio (CR):

$$CR=\frac{CI}{RI}$$

where RI is the Random Index, determined experimentally based on matrix size.

The AHP is useful for solving unstructured problems in social sciences, but in some studies, it is difficult to ensure the independence of survey items [72,73]. Therefore, a consistency ratio of 0.2 or below is considered acceptable. Additionally, if the consistency ratio is below 0.1, the resulting weights are considered logically consistent and are deemed reasonable. In this study, results with a consistency index of 0.2 or below were considered and all responses were regarded as logically consistent. The details of the consistency index are shown in Table 6.

Prior to determining the priority ranking of each factor using AHP analysis, the respondents’ weights are consolidated through the geometric mean to obtain an overall weight. To achieve this, an aggregated geometric mean matrix is derived by combining the pairwise comparison matrices of 29 respondents who satisfied the consistency ratio threshold of 0.2 or lower. This aggregated matrix is then utilized to calculate the weights for key performance indicators related to service robotics in senior community. The findings from the pairwise comparison matrix, which illustrate the relative importance of each evaluation criterion in this study, are presented in Table 7.

The measurement results of the relative importance and priority ranking for the seven evaluation domains—Technical Performance, User-Centered Performance, Social and Psychological Impact, Ethical and Safety Performance, Economic and Operational Performance, and Service Efficiency—within the two-tier AHP model are presented in Table 8. The relative importance and priority ranking among the evaluation domains are as follows: Technical Performance (0.256), User-Centered Performance (0.205), Social and Psychological Impact (0.167), Ethical and Safety Performance (0.139), Economic and Operational Performance (0.126), and Service Efficiency (0.105).

## 5. Discussion

This study analyzed the performance evaluation of service robotics not merely as a measurement of technological performance but through a multidisciplinary and governance-based approach using a hierarchical structure (the AHP model). The objective was to establish a theoretical foundation for standardizing service robotics key performance indicators in senior communities, which could also be applicable to other public sectors.

This study contributes academically by employing a Mixed Methods Research (MMR) approach, integrating qualitative (Focus Group Interviews, FGIs) and quantitative (AHP) analysis. Unlike previous studies relying on single methodologies, it first derives key concepts through expert interviews and then verifies indicator importance via hierarchical analysis. This framework can guide future research on technology-based elderly care and smart care systems. Moreover, while prior research on service robotics has primarily emphasized technical performance and economic efficiency, this study introduces a comprehensive performance evaluation model that integrates social and psychological aspects, ethical and safety considerations, and user experience. The high priority given to technological performance and user-centered performance highlights the crucial role of not only technical capabilities but also user experience and technology acceptance in successfully integrating robotics into senior communities.

The AHP analysis results indicate that Technological Performance (0.256) was the most important factor, followed by User-Centered Performance (0.205) and Social and Psychological Performance (0.167). This finding implies that service robotics in senior communities cannot achieve successful adoption by focusing solely on functional efficiency. Therefore, improving user-friendly interfaces (UI/UX) is essential to ensure accessibility for older adults. This can be achieved by enhancing features such as simplified menus, voice guidance, and optimized touchscreen functions [74]. Additionally, social robots should be utilized to promote emotional connection and psychological stability, incorporating technologies such as facial expression recognition, sentiment analysis, and conversational AI to facilitate more natural human–robot interactions [75].

The relatively high importance assigned to Ethical and Safety Performance (0.139) compared to Economic and Operational Performance (0.126) suggests that factors such as safety, privacy protection, and ethical design must be prioritized in the implementation of service robotics. Since older adults may face challenges such as cognitive decline, concerns about data privacy, and increasing reliance on technology, ensuring safety and ethical compliance should take precedence over mere operational efficiency [76]. Additionally, an emergency response system should be established to detect unforeseen situations (e.g., falls and health anomalies) and immediately notify caregivers or medical professionals. This system should include IoT-based real-time monitoring and emergency alert features.

Finally, while Economic and Operational Performance (0.126) and Service Efficiency (0.105) received lower importance rankings, optimizing service delivery and ensuring economic sustainability remain crucial for the successful implementation of service robotics. The adoption of a subscription-based robotic service model can help reduce initial investment costs and offer greater flexibility in utilization through rental/subscription options. Moreover, establishing a hybrid operational model in which robots collaborate with medical professionals and caregivers can enhance service efficiency by enabling robots to assist in basic tasks while human caregivers handle more complex responsibilities.

## 6. Conclusions

The findings of this study provide a foundational reference for the development of sustainable smart elderly care services. While Technological Performance was identified as the most critical factor, the significant importance assigned to Social and Psychological Performance and Ethical and Safety Considerations highlights the necessity of balancing technological innovation with social value realization in future service robotics development. This underscores the growing relevance of inclusive technology, social robotics, and ethical AI in shaping future research directions [77].

This study suggests that service robotics should not be perceived solely as a technological innovation but as a comprehensive system that integrates user experience, safety, operational efficiency, and sustainability. Accordingly, human-centered design, safety assurance, economic efficiency optimization, and public–private collaboration models are essential for practical implementation. To ensure the effective deployment of service robotics in senior communities, a multi-faceted approach encompassing technological advancements, policy support, operational model enhancements, and continuous user feedback must be adopted.

This study used expert FGIs and AHP to derive indicators, but lacks input from older adults and empirical evidence. Furthermore, its applicability to different cultural, social, and policy contexts remains uncertain as the research was conducted in South Korea.

To address these limitations, future research should refine service robotics performance indicators by incorporating factors such as technology acceptance, social perceptions, policy frameworks, and cultural influences. Additionally, empirical studies using AI and big data-driven assessments are needed to quantitatively analyze the long-term impact of service robotics. Furthermore, user acceptance research should examine older adults’ actual responses and adoption behaviors to validate and refine the performance framework.

## Figures and Tables

**Table 1:** Mixed Methods Research (MMR) process.

Phase	Stage	Key Activities
Phase 1 Qualitative Research	FGI Preparation	- Define research objectives; - Design questions and research methodology; - Recruit and select interview participants; - Send email notifications regarding research objectives and FGI procedures.
	1st FGI	- Introduction to service robotics; - Discussion and exchange of opinions on service robotics in senior communities.
	2nd FGI	- Exploration of key performance indicators for service robotics in senior communities; - Classification of key performance indicators.
	3rd FGI	- Derivation of key performance indicators (KPIs) for service robotics; - Definition of indicators and collection of additional feedback.
Phase 2 Quantitative Research	AHP Preparation	- Define research objectives; - Develop the survey questionnaire; - Recruit survey participants (senior community stakeholders, service robotics experts from academia, industry, and government).
	AHP Survey	- Distribute and collect 32 survey responses via email; - After coding and cleaning, 29 valid questionnaires were analyzed.
	AHP Data Analysis	- Utilize Social Science Research Automation (SSRA) cloud service; - Conduct AHP of service robotics key performance indicators.

**Table 2:** FGI panel composition.

Field	Affiliation	Position	Experience	Remark
A	Senior Welfare, ** University	Professor	25 years	Panel #1
	Regional Policy, ** Research Institute	Research Manager	8 years	Panel #2
	Business Planning, **** Senior Residence	Developer	13 years	Panel #3
B	Computer Engineering, *** University	Professor	11 years	Panel #4
	Technology Commercialization, ** Province Office	Service Planner	22 years	Panel #5
	AI and Data Science, ** University	Senior Researcher	10 years	Panel #6

A: Gerontology and Social Welfare, B: Robotics Engineering and Technology Management. *: All FGI participants provided individual consent for participation; therefore, their institutional names are represented by asterisks (*) corresponding to the number of initials in each name.

**Table 3:** Demographic characteristics of AHP respondents.

Category		Number (n)	Percentage (%)
Gender	Male	19	65.5
	Female	10	34.5
Age	40 and under	6	20.7
	40s	10	34.5
	50s	11	37.9
	60 and above	2	6.9
Occupation	Professor	8	27.6
	Researcher	3	10.3
	Government Official	4	13.8
	Public Institution Staff	3	10.3
	Service Robotics Practitioner	4	13.8
	Senior Community Practitioner	5	17.2
Industry Fields *	Human Health and Social Work Activities	8	27.6
	Administrative and Support Service Activities	6	20.7
	Information and Communication	4	13.8
	Professional, Scientific, and Technical Activities	6	20.7
	Manufacturing and Engineering	5	17.2
Total		29	100

*: The industry fields are categorized based on ISIC (International Standard Industrial Classification) developed by the United Nations (UN).

**Table 4:** Senior community-based service robotics key performance indicator development: categorization through FGIs.

Categorization	Key Transcriptions	Remark
Technical Performance	“the robot works should work well without error”	Panel #4
	“navigation performance is the most important. especially for the senior community, the ability to avoid obstacles in narrow spaces is essential”.	Panel #1
	“sensors recognize the surrounding environment while working properly”	Panel #3
	“AI-based learning skills need to be improved. As interactions with the user are repeated, the robot must recognize the pattern and adapt automatically”.	Panel #6
	“be easy to fix when it breaks down”	Panel #5
User-Centered Performance	“the button is simple for the elderly to use, and the operation should be easy”	Panel #3
	“ease of use is the key. A simple interface and clear voice guidance are needed for seniors to use”.	Panel #2
	“even those with physical disabilities use it easily”.	Panel #1
	“need a personalization system that can adjust the function to the user’s needs”.	Panel #4
Service Efficiency	“finish the work well on time without any problems”	Panel #3
	“Slow response can lead to poor reliability. Rescue is needed to respond quickly, especially in emergency situations”.	Panel #2
	“work well all day, or for the time needed for the service“	Panel #5
	“While robots are taking over the job, employees can focus on more important interpersonal services”.	Panel #6
Economic and Operational Performance	“Even if initial costs are high, low maintenance costs should be beneficial in the long run”.	Panel #3
	“the cost of electricity and maintenance should be appropriate”.	Panel #4
	“based on the ROI, efficient results should be achieved”.	Panel #5
	“Scalability is also important. Software integration is needed to make it easier for one robot to operate in other facilities”.	Panel #6
Social and Psychological Impact	“Seniors feel lonely a lot. For a robot to play an emotional role, it needs emotion recognition and empathy”.	Panel #1
	“Robots need to become not just machines that perform tasks, but helpers that promote social interaction”.	Panel #2
	“even if the robot approaches, it should not be anxious and feel friendly”.	Panel #3
	“improve the quality of life of the elderly”	Panel #5
Ethical and Safety Considerations	“Privacy is essential. In particular, information related to healthcare requires encryption storage and access control”.	Panel #4
	“ethical standards must be established and complied with when interacting with the elderly”.	Panel #2
	“In the event of an emergency, the robot should be able to recognize it quickly, take appropriate action, or ask for help from the outside”.	Panel #3
	“A regular inspection system should be in place to ensure that the service robot complies with legal regulations”.	Panel #1

**Table 5:** Operational definitions of key performance indicators.

Key Performance Indicators	Operational Definitions
Technical Performance	Evaluates the overall technological completeness of service robots by assessing performance reliability, environmental adaptability, autonomy, and ease of maintenance.
User-Centered Performance	Assesses user-centered design by considering physical and cognitive accessibility, interface intuitiveness, operational convenience, and personalized customization features.
Service Efficiency	Measures service performance based on operational accuracy, continuity, resource optimization, and emergency response capabilities.
Economic and Operational Performance	Comprehensively evaluates the economic and operational feasibility of service robots, including total cost of ownership (TCO), cost reduction effects, operational sustainability, and technological scalability.
Social and Psychological Impact	Analyzes the social and psychological impact of service robots in senior communities, focusing on social relationship formation, emotional stability, cognitive health support, and overall life satisfaction.
Ethical and Safety Considerations:	Evaluates the ethical and safety aspects of service robots based on data security, compliance with ethical protocols, risk management systems, and legal and regulatory adherence.

**Table 6:** Consistency index.

Item	λ-max	C.I.	C. Ratio
Goal	6.02025	0.00405	0.00327

**Table 7:** Aggregated geometric mean matrix.

	TPE	UCP	SPI	ESP	EOP	SEF
TPE ^a^	1.000	1.375	1.511	1.843	2.035	2.258
UCP ^b^	0.727	1.000	1.143	1.838	1.666	1.799
SPI ^c^	0.662	0.875	1.000	1.252	1.167	1.596
ESP ^d^	0.543	0.544	0.798	1.000	1.238	1.484
EOP ^e^	0.491	0.6	0.857	0.808	1.000	1.237
SEF ^f^	0.443	0.556	0.626	0.674	0.808	1.000

^a^: Technical Performance, ^b^: User-Centered Performance, ^c^: Social and Psychological Impact, ^d^: Ethical and Safety Performance, ^e^: Economic and Operational Performance, ^f^: Service Efficiency.

**Table 8:** Weights and priority ranking by indicator.

Category	Subcategory	Importance (%)	Ranking
Service Robotics	Technical Performance	0.256	1
	User-Centered Performance	0.205	2
	Social and Psychological Impact	0.167	3
	Ethical and Safety Performance	0.139	4
	Economic and Operational Performance	0.126	5
	Service Efficiency	0.105	6

## Data Availability

Data are contained within the article.
